# Association between Liver and Kidney Function and Birth Outcomes in Pregnant Surinamese Women Exposed to Mercury and Lead in the Caribbean Consortium for Research in Environmental and Occupational Health (CCREOH) Environmental Epidemiologic Cohort Study

**DOI:** 10.3390/jox14030059

**Published:** 2024-08-01

**Authors:** Sheila Kort, Jeffrey Wickliffe, Arti Shankar, Hannah H. Covert, Maureen Lichtveld, Wilco Zijlmans

**Affiliations:** 1 Faculty of Medical Sciences, Anton de Kom University of Suriname, P.O. Box 9212 Paramaribo, Suriname; wilco.zijlmans@uvs.edu; 2 Department of Environmental Health Sciences, School of Public Health, University of Alabama at Birmingham, Birmingham, AL 35294, USA; jkwickli@uab.edu; 3 Department of Biostatistics and Data Science, School of Public Health and Tropical Medicine, Tulane University, New Orleans, LA 70112, USA; sarti@tulane.edu; 4 Department of Environmental and Occupational Health, School of Public Health, University of Pittsburgh, Pittsburgh, PA 15261, USA; hcovert@pitt.edu (H.H.C.); mlichtve@pitt.edu (M.L.)

**Keywords:** heavy metals, pregnant women, liver function, kidney function, gestational age, birthweight, apgar score

## Abstract

Exposure to mercury (Hg) and lead (Pb), in combination with liver and kidney impairment, may result in adverse birth outcomes. From 408 women in the age range of 16 to 46 years, living in rural and urban areas in the interior of Suriname, we looked at the association between adverse birth outcomes and exposure to Hg and Pb in combination with liver and kidney function. This group of women represented a subcohort of pregnant women who participated in the Caribbean Consortium for Research in Environmental and Occupational Health (CCREOH)—Meki Tamara study. Liver function was assessed by measuring aspartate amino transferase (AST), alanine amino transferase (ALT), and gamma-glutamyl transferase (GGT). Kidney function was assessed by measuring creatinine, urea, and cystatin C. We defined preterm births as birth before 37 weeks of gestation, low birthweight as birthweight < 2500 g, and low Apgar score as a score < 7 at 5 min, and these were used as indicators for adverse birth outcomes. Small size for gestational age was defined as gestational age < −2SD weight for GA. We found significant statistical associations between biomarkers for liver and kidney functions and adverse birth outcomes Apgar score and gestational age. No significant association was found between heavy metals Hg and lead and adverse birth outcomes.

## 1. Introduction

Adverse birth outcomes are a global health problem, including preterm birth, low birthweight (LBW), and prenatal asphyxia reflected by low Apgar score. The subsequent effects of adverse birth outcomes (ABOs) are various, and it has been shown that preterm and low-birthweight children are at greater risk for future mortality and (neuro)developmental problems [1,2]. Low Apgar scores are also negatively associated with risk of neurodevelopmental delay [3] and low Apgar scores are associated with infant mortality, even for low-risk pregnancies [4,5]. Estimates are that 27% of livebirths in low-income and middle-income countries are infants small for gestational age (SGA) [6]. Globally, 15.5% of all newborns are born with LBW, of which 96% are born in developing countries [7].

Causes of ABOs can be divided into various categories, such as behavioral and psychosocial [8], sociodemographic [9,10], medical, and environmental [9]. ABOs have been associated with environmental pollutants such as air pollution [10,11], agricultural chemicals [12], and heavy metals [13,14,15]. Maternal exposure to heavy metals such as mercury and lead may have adverse effects on both the mother and the unborn child. Prenatal exposure to mercury can negatively affect birthweight, Apgar score, and preterm birth [15,16,17]. 

Impaired liver or kidney function in pregnant women may also lead to adverse birth outcomes.

An Indian study showed that pregnant women with chronic kidney disease (CKD) had higher incidence of adverse events, like pre-eclampsia, low Apgar score, and small size for gestational age, and a Chinese study showed that liver dysfunction during pregnancy was associated with an increased risk of preterm birth [18,19].

Liver disease can affect pregnancy and its outcome. This is also the case with kidney disease [20,21]. There are various liver diseases which are unique to pregnancy, such as pregnancy-induced elevated liver enzymes, (pre-)eclampsia, and HELLP syndrome (Hemolysis Elevated Liver function and Low Platelet count) [20]. The association of liver disease with adverse pregnancy outcomes remains unclear; however, there are indications that liver impairment may cause an increased risk of preterm birth [19,22]. Women with kidney disease have a higher chance of delivery through a cesarean section or preterm birth [22]. Depending on the severity of kidney function impairment in pregnant women, ABOs may occur more frequently and may be more severe. The risk of ABOs increases with the stage of kidney disease [18,21]. Low Apgar scores were higher in patients with later stages of chronic kidney disease [18].

Exposure to mercury may lead to kidney disease [23] and is also associated with an elevated concentration of gamma-glutamyltransferase, a liver function biomarker, indicating possible liver impairment [24]. Cave et al. showed an unexplained ALT elevation associated with polychlorinated biphenyl PCB, lead, and mercury exposure in adult participants for the National Health and Nutrition Examination Survey (NHANES) [25]. In another study, amino acid profiles, alkaline phosphatase, and lactate dehydrogenase were significantly higher in industry workers exposed to lead, indicating possible liver impairment [26]. A metastudy in the general public found supportive evidence of a negative influence of Hg exposure on the risk of chronic kidney disease. In the same study, factors such as the type of Hg (inorganic, organic), the duration and dose of exposure, the gender, and age of the exposed population may influence the risk of chronic kidney disease [27]. In pregnant women, it has been shown that high blood lead levels (BLLs) and blood mercury levels may also affect the mother’s liver and kidney function [24,28,29]. A review article by Orr et al. described a direct relationship between Pb exposure and the development of chronic kidney disease, and importantly, they mention changes at a cellular level in the kidneys, even if renal function is not compromised [30].

In Suriname, a country situated on the northeastern coast of South America, a 2020 study reported adverse outcomes, including preterm birth (15.1%), small size for gestational age (1.2%), low birthweight, and low Apgar score (3.8%) [31]. The prevalence for LBW in South America was 8.8% and 11.7% in the Caribbean [32]. In Suriname, 90% of the 590,549 population lives in the capital Paramaribo and the coastal area. The remainder live in the tropical rainforest interior (90% of the country’s landmass). Artisanal and small-scale gold mining (ASGM) is a major contributor to mercury contamination, especially in the interior of Suriname. People working in ASGM and those living in surrounding communities are potentially exposed to high levels of mercury and exposure of residents is mainly through the consumption of highly contaminated fish in the ASGM areas [33]. Various sources of lead exposure include lead-based paint (still present in older buildings), gasoline (although banned), and traces of lead in groundwater [34]. In the neighboring country of French Guiana, lead exposure through manioc consumption is believed to be important. Since the indigenous people in Suriname also commonly cultivate and consume manioc, lead exposure through manioc consumption may also be important in Suriname [35].

The Caribbean Consortium for Research in Environmental and Occupational Health (CCREOH) is an environmental epidemiology cohort study of 1200 mother/child dyads living in the urban and interior regions of Suriname. In the CCREOH cohort study, exposure to a complex mixture of elements and toxicants was examined, and other chemicals in these pregnant women were assessed in association with adverse birth outcomes [36,37]. In addition to lead and mercury, cadmium (Cd) levels were analyzed, because of potential exposure due to tobacco smoke and municipal waste incineration. Exposure to pesticides in rural areas was the reason for measuring manganese (Mn) and tin (Sn). The traditional practice in women of African descent of consuming clay (pemba) during pregnancy and use of aluminum cooking ware are potential sources of aluminum exposure. Selenium (Se), an antioxidant found in nuts, is a possible protective agent against exposure to heavy metals. Iron has been included for further research into anemia within the CCREOH cohort. The measurement of all these elements reflects the actual exposure to multiple elements while living in Suriname. Due to proven high exposure to mercury (artisanal gold mining, eating contaminated fish) and lead (eating manioc and wild game) in Suriname, we choose to mainly focus on Hg and Pb in this study, while briefly mentioning the other elements.

Little is known about the effects of liver and kidney functions and adverse birth outcome in a population with high exposure to Hg and Pb. The goal was to develop a predictive model for various birth outcomes using blood metals, kidney, and liver functions as independent parameters, adjusted for sociodemographic variables. In this study, we assess the effects of prenatal exposure to Hg and Pb and potential impaired liver and kidney function on ABOs (preterm birth, low birthweight, and low and Apgar score) in a subcohort of pregnant women enrolled in the CCREOH cohort study. 

## 2. Materials and Methods

### 2.1. Study Population

As described in our previous paper [28], a total of 1200 pregnant women were recruited between December 2016 and July 2019. Inclusion criteria included age between 16 and 45 years with singleton gestation and speaking Dutch, Sranan Tongo, Sarnami, Saramaccan, or Trio. They had to planned to deliver at one of the following study hospitals or study sites: (1) Paramaribo, at four hospitals (the Academic Hospital Paramaribo, Diakonessen Hospital, ‘s-Lands Hospital, Saint Vincentius Hospital) and at prenatal clinics and midwife facilities of the regional health department; (2) Nickerie, at the Mungra Medical Centre Hospital and at regional health department clinics and facilities; and (3) the Amazonian interior, at multiple health care clinics of the Medical Mission Primary Health Care Suriname. All women provided written informed consent/assent. Blood collection, carried out through standard venipuncture into sterile vacutainers by trained health professionals, was conducted at two time points (late first/early second and third trimester) during pregnancy. Whole blood samples were processed and stored frozen at −80 °C in the Clinical Chemistry Laboratory of the Academic Hospital in Paramaribo, Suriname. A total of 1200 women were recruited, of whom 1189 were followed up. We measured metal and element concentrations in blood samples collected during the late first trimester/early second trimester in a subset of 400 women selected from the larger cohort. From 408 women, we measured the liver and kidney function.

### 2.2. Metals and Elements

Four hundred frozen blood samples were analyzed at the Wisconsin State Laboratory of Hygiene Trace Element Research Laboratory. Eight target elements (Al, Cd, Fe, Hg, Mn, Pb, Se, Sn) were quantified by SF-ICPMS (ThermoFinnigan Element 2XR; Thermo Fisher, Waltham, MA, USA). Standard reference materials, matrix spikes, and method duplicates were used for quality assurance and quality checking (QA/QC). All internal and external QAs/QCs were acceptable. The project-specific 3-sigma method detection limits calculated from a meta-analysis of low-level standards run across all 17 analytical batches were: Al: 1.1 µg/L; Mn: 0.07 µg/L; Fe: 42 µg/L; Se: 2.1 µg/L; Cd: 0.006 µg/L; Sn: 0.04 µg/L; Hg: 0.05 µg/L and Pb: 0.06 µg/L.

### 2.3. Liver and Kidney Biomarkers

Liver function was measured by Aspartate Amino Transferase (AST), Alanine Amino Transferase (ALT), and Gamma-Glutamyl Transferase (GGT). Kidney function was analyzed using Creatinine (Cr), Urea (Ur), and Cystatin C (CysC) measured in aDXC-600 Beckman Coulter Analyzer (Synchron systems). Reference ranges for liver functions used are AST 15–41 U/L; ALT 7–52 U/L; and for GGT, 7–50 U/L.

For adult females, the creatinine level reference range is 44–97 μmol/L. The reference range for urea in blood or serum is 1.8–7.1 mmol/L, and for CysC, it is 0.57–1.79 µg/mL.

In pregnant women, the reference ranges are in the lower ranges of the non-pregnant reference levels, with the lowest levels in the third trimester [38].

### 2.4. Birth Outcomes

Information about birth outcomes was obtained by recruiters from hard copy delivery books completed by midwives at the facility where the baby was born. This information was transferred and managed using REDCap (Research Electronic Data Capture), a secure, web-based software platform designed to support data capture for research studies [39,40]. Adverse birth outcomes were defined as follows: preterm births as birth before 37 weeks of gestation, low birthweight as birthweight < 2500 g, and low Apgar score as a score < 7 at 5 min. We also looked at small size for gestational age (SGA), defined as <−2SD weight for gestational age.

### 2.5. Data Analysis

The Kolmogorov–Smirnoff test as well as analysis of marginal residuals indicated skewed distribution for all three birth outcomes, namely gestational age, birthweight, and Apgar score. The Levene test indicated homoscedasticity violations as well. The variables were therefore summarized using medians and interquartile ranges. Given the distribution of the outcome variables and violations of homoscedasticity, weighted least square regression analysis was used to develop predictive models for each of the three outcome variables. Each predictor was adjusted for the other. The models were tested for multi-co-linearity between the predictor variables using a variance inflation factor greater than 1.5. Selection of predictors was based on in-depth literature review [19,21,22,24,26]. All analysis was performed using SPSS version 27 at the 5 percent level of significance [41].

### 2.6. IRB

This study was approved by the Institutional Review Board (IRB) of Tulane University (839093) and the Medical Ethical Commission of Suriname’s Ministry of Health (VG 023-14). All included women (18+) provided written informed consent, and assent was obtained from women who were 16 or 17 years old.

## 3. Results

Table 1 shows the distribution of participants’ sociodemographic characteristics based on region of residence, maternal age at intake, ethnicity, and education. Most participants (74.5%) lived in urban areas (Paramaribo, Wanica, Commewijne, Saramacca, and Para), while the lowest proportion (11.8%) were from interior districts, (Marowijne, Brokopondo, and Sipaliwini). Participants 20–34 years of age were most represented, while the group between 16 and 19 years had the lowest representation. Women of African descent represented 46.8% of the participants and women of Asian descent had a representation percentage of 25.5%. Regarding education, most women had an upper secondary, vocational, or tertiary education. 

All liver and kidney function measurements fell within the reference ranges (Table 2). However, median blood mercury and blood lead levels were higher than the internationally accepted safe threshold. The WHO states that no level of lead is safe and the USCDC uses a blood mercury level of 3.5 µg/L as the action level for children [42,43].

**Figure 1:**
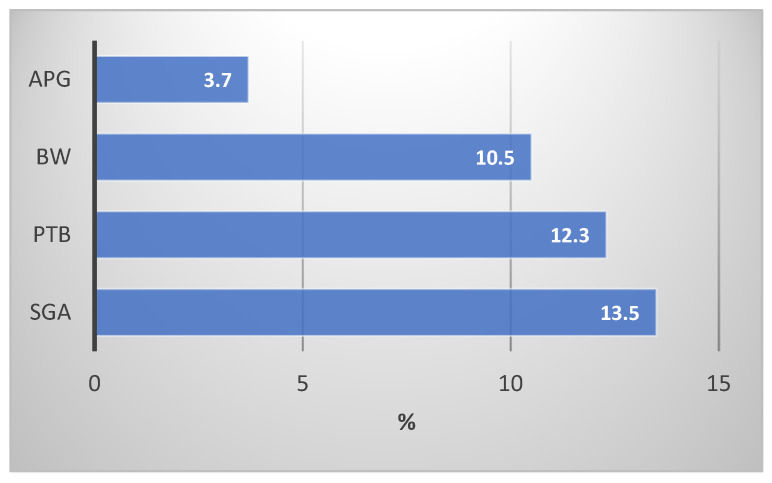
Percentage of adverse birth outcomes per category. APG = Apgar score < 7 at 5 min; BW = birthweight < 2500 grams, PTB = preterm birth < 37 weeks; SGA GA < −2SD.

Figure 1 shows the percentages of adverse birth outcomes. Small size for gestational age had the highest incidence of 13.5% (*n* = 55), followed by preterm birth 12.2% (*n* = 50), and low birthweight 10% (*n* = 43); low Apgar score was seen in 3.7% (*n* = 15) of the cases.

## 4. ABOs in Relation with Liver and Kidney Function and Blood Hg and Pb Levels

Results from Table 3 show that gestational age is statistically significantly associated with kidney function, as measured by Cystatin C (B = 1.41, *p* = 0.0056), and liver function, as measured by blood ALT (B = 0.18, *p* ≤ 0.0001) and AST (B = −0.111, *p* = 0.0004).

Statistically significant differences in gestational age were also found in babies born in the interior versus those born in urban areas (B = 2.13, *p* ≤ 0.001) and for babies born to women in the age category 16–19 and 35+ and babies born to women in the age category 20–34. (B = −1.33, *p* ≤ 0.001. B = −1.28, *p* = 0.0005). 

Blood Mn (B = 0.051, *p* < 0.0001), Fe (B ≤ 0.001, *p* = 0.0001), and Cd (B = 5.454, *p* < 0.0001) levels were all positively associated with gestational age. Higher levels of these metals were associated with increased gestational age and lower blood Se levels (B = −0.025, *p* < 0.0001). We ran logistic regression for SGA. The beta coefficients and odds ratio did not show any significant correlation between SGA and biomarkers for liver and kidney functions or any metals and elements. Also, there was no correlation between SGA and any demographic parameters mentioned in this paper (see Supplement Table S1).

Birthweight was not statistically significantly associated with any liver or kidney function measurements or with blood Hg or blood Pb levels. However, statistically significant associations were found between birthweight and participants in the various regions. Participants from rural areas had significantly lower birthweights compared to participants in the reference group urban area (B = −0.2, *p* = 0.0006). Birthweight was also significant within the maternal age categories and ethnicity. The birthweights of babies of women aged 16–19 years were significantly lower than of women in the age group 20–34 years (B = −0.108, *p* = 0.052), and women with an Asian ethnic background had babies with significantly higher birthweights than women of African descent (B = 0.13, *p* = 0.0239). An increased birthweight was also associated with increased blood Al levels (B = 0.00048, *p* = 0.0392).

An increase in Apgar score was associated with an increase in liver function, as measured by ALT (B = 0.042, *p* = 0.0441), and kidney function, as measured by Urea (B = 0.427, *p* = 0.0024). An increasing Apgar score was statistically associated with a decreasing liver function, as measured by AST (B = −0.060, *p* = 0.0035), and decreasing kidney function, as measured by Cystatin C (B = −0.640, *p* ≤ 0.0001). As for associations for additional added metals, we found that Apgar score was not statistically significantly associated with Hg and Pb, but higher Apgar score correlated with lower Al levels (B = −0.0169, *p* = 0.0041) and Fe levels (B = −0.00000358, *p* < 0.0001). There were statistically significant associations between Apgar score and the demographic parameters. Participants from the rural areas had babies with significantly lower Apgar scores compared to participants in the reference group urban area (B = −1.03, *p* < 0.0001). Apgar scores were significantly higher for women aged 16–19 years compared to women aged 20–34 years (B = 0.666, *p* = 0.001). Babies born to women with a secondary level education had significantly lower Apgar scores compared to women with none or primary education (B = −0.477, *p* = 0.0003). Table 3 shows the weighted regression results for birthweight, gestational age, and Apgar score. Three multiple regression models were run, so there is a probability of type 1 error inflation. Using the Bonferroni adjustment gives us an adjusted significance level of 0.01. The only result that will be affected is the effect of blood aluminum on birthweight, which will no longer be significant.

## 5. Discussion

In this study, we examined the association between biomarkers of liver and kidney function, heavy metal exposure, and ABOs. We found statistically significant associations between birth outcomes, elements Al, Mn, Fe, and Cd, and the parameters for liver and kidney function. The percentages of adverse birth outcomes that we found are in line with the Surinamese population. One study in 18.290 women from 2016–2017 showed a LBW of 15.1%, preterm birth (GA < 37 weeks) of 14%, and a low Apgar score of 3.9%. A case–control study conducted in 2017–2018 between women with and without hypertensive disorder and the birth outcomes from women without hypertensive disorder showed a preterm birth of 12.2%, LWB of 12.7%, and a low Apgar score of 3.3% [31,44]. In comparison, LWB for women in the USA in 2017 was 2.6% amongst all ethnicities, with the highest rate of 4.3% for black non-Hispanic women and the lowest rates between 2.0 and 2.2% for white non-Hispanic women, Mexican women, and Hispanic women [1]. In Ethiopia, the incidence of preterm birth was 14.3% and 11.2% for LWB [45].

Gestational age went up with higher liver ALT and kidney Cystatin C markers, whereas GA went down with higher liver AST. Birthweight had no significant association with kidney or liver function biomarkers. Apgar score had a significant positive association with liver ALT and blood urea, meaning that a high Apgar score was associated with higher ALT and blood urea levels. In contrast, a higher Apgar score was significantly associated with decreasing liver AST and kidney Cystatin C levels. There were no significant associations found between SGA and any of the metals, elements, or demographic parameters. 

In this study, none of the adverse birth outcomes were statistically significantly associated with Hg and Pb levels, but statistically significant associations were found with Al, Mn, Fe, and Cd levels.

During pregnancy, it is common practice to measure certain blood parameters where reference intervals may differ from those taken before or after pregnancy. Dai et al. showed that kidney function biomarkers creatinine and Cystatin C in a Chinese cohort dropped during pregnancy [38]. A meta-analysis by Lopes van Balen et al. showed a decrease in Glomerular Filtration Rate and a decrease in serum creatinine during a healthy uncomplicated pregnancy, but serum creatinine did not change with gestational age [46]. Harville et al. also did not find strong evidence between gestational age and kidney function [47].

There is a significant positive association between gestational age and Cystatin C in our study, with the gestational age going up with higher Cystatin C levels. Other studies show that kidney function, measured as Glomerular Filtration Rate, and creatinine decreased with higher gestational age [48]. An explanation for this difference could be the timing of taking the sample, which can lead to a negative or positive association. According to Harel et al., Cystatin C levels decrease during the first trimester and increase during the third trimester [49].

Liver function biomarkers ALT and AST show different alterations during pregnancy. Some studies show either an increase, decrease, or fluctuation within reference ranges for these biomarkers during pregnancy. Most of the time, the fluctuations are physiologically and gestation-related. In some cases, pathological causes like hepatocellular disease or biliary disease may cause elevated AST and ALT levels [38,50]. In our study, this phenomenon was also noticed, where gestational age went down with higher AST and went up with higher ALT.

No association was found between gestational age and either mercury or lead exposure. The same results were found in a Saudi Arabian population, where neither lead nor mercury exposure was associated with the birth outcome parameters gestational age and preterm birth [15]. We found a significant association, where higher Cd levels are seen with higher gestational age (B = 5.454, *p* < 0.0001), which is contrary to the findings of Johnston et al., who showed an inverse association between cadmium and birthweight and an increased chance of infants being born small for gestational age [51]. Al-Saleh et al. also found various significant associations between umbilical cord cadmium levels, low Apgar score, and gestational age. Maternal blood did not show these associations [15]. In Myanmar, women with prenatal cadmium exposure had a higher incidence of low birthweight [13]. Reasons or explanations for why our study did not follow the same trend could be found in the fact that there are more complex mechanisms involved.. The suggestion that placental Metallothionein may play a role in protecting the fetus from Cd toxicity by binding to this metal may be just one of many reasons for our findings [52].

There are regional variances in Pb and Hg reference concentrations. We compared various reference values for Pb and Hg exposure in women and found a variety of outcomes. A Finnish study reported levels for Pb and Hg of 1.23 µg/dL and 2.20 µg/L, respectively [53]. The NHANES study reported levels of 0.64 µg/dL for Pb and 0.63 µg/L for blood Hg levels [54]. German reference values for blood Pb and mercury were set to 0.7 µg/dL and 2.0 µg/L, and the Korean National Health and Nutrition Examination Survey (KNHANES) reported blood lead levels of 2.29 µg/dL and blood mercury levels of µg/L in women [55,56]. These variations make it difficult to compare our results. Because of the environmental impact and effect on offspring, we chose to use the lowest reference levels. For Pb, these are the WHO levels, stating that no level of Pb is considered safe, and for blood mercury, we use the U.S. Environmental Protection Agency (EPA) level of 0.60 µg/L [42,57].

This study does not show any significant association between birthweight and kidney function biomarkers, liver function biomarkers, or mercury or lead levels. Most studies demonstrate that only defined kidney impairment has an effect on birthweight and other adverse birth outcomes [18,22]. However, in this study, all kidney biomarker levels were within the reference range, suggesting no severe kidney impairment.

We found significant associations between Apgar scores and kidney function biomarkers. Increasing Apgar scores were associated with higher blood urea levels and with lower blood Cystatin C. Women with chronic kidney disease (CKD) showed an increase in the number of babies born with low Apgar scores [18]. In our study, the kidney function biomarkers were within reference ranges, but higher Apgar scores were associated with higher blood urea levels and low Cystatin C levels. 

Our results show an association between Apgar scores and the liver function biomarkers ALT and AST. Apgar scores went up with higher ALT and lower AST. Factors not included in this study, such as maternal health, use of drugs, pre-eclampsia, and diseases such as gestational diabetes, are only a few of the many causes of variation in liver function indicators [58,59].

There was no statistically significant association between Apgar scores and Hg or Pb levels, suggesting that these heavy metals may not have an effect at these concentrations. For the other metals and elements included in this study, we found statistically significant positive associations where gestational age went up with higher levels of the elements Mn, Fe, and Cd and lower levels of Se. Increasing birthweight was associated with increasing Al levels, and increasing Apgar score was associated with decreasing Al and Fe levels.

Results from the National Children’s Study (NCS) in the United States suggested that prenatal exposure to the elements Cd, Se, Mn, and Pb may influence birth outcomes such as gestational age, birthweight, and head circumference [60]. In a Saudi Arabian population of pregnant women, birthweight, SGA, and Apgar score were influenced by Cd in umbilical cord blood levels [15]. However, there is a range of different findings when it comes to exposure to metals associated with SGA. Thomas et al. found no association between Cd and lead and risk for SGA, and a meta-analysis by Sezavar et al. in 2021 concluded that there is an indication that maternal blood Cd exposure is associated with SGA, but results differ per study, as gender of neonates and ethnic diversity may be of influence [61,62]. An increased risk for SGA was observed with maternal mercury exposure in a population of Canadian pregnant women [61]. And in a Taiwanese cohort, exposure of mothers to lead particles in the air was associated with an increased risk of SGA in their offspring [63]. The suggestion that SGA is influenced by gender of the neonates or ethnicity may also be the case in our study.

It is also suggested that an interaction between Hg and Se may be a measure for explaining Hg-induced adverse birth outcomes [64].

Our findings of variation in birth outcomes compared to other studies suggest the influence of additional factors, potentially internal biochemical mechanisms or external factors like diet and habits. These factors might have synergistic (compounding) or protective effects. The interaction between maternal mixed metal exposures and adverse birth outcomes appears to be more complex than previously understood. A literature review did not provide clear explanations for these discrepancies, necessitating speculation and further investigation into the potential reasons behind our observations. This complexity underscores the need for additional studies to clarify the complexity of interactions between mixed metal exposure and birth outcomes.

## 6. Limitations

Socio-economic factors, such as availability, wide usage of these parameters, and being covered by insurance and limited sources in LMIC were our considerations in choosing the liver and kidney function biomarkers. We measured the most common and most accessible clinical liver function biomarkers. We were unable to measure more uncommon or less accessible markers in a low- and middle-income country such as Suriname, e.g., Anti-Mitochondrial Antibodies (AMAs) and 5’-Nucleotidase, and experimental markers such as hepatokines and markers for oxidative stress, which can identify liver damage at an earlier stage. For kidney function, β2-microglobulin may be an additional marker to assess kidney impairment. Due to physiological changes, the time during pregnancy at which blood is drawn may have an effect on the parameters [38]. During pregnancy, the blood levels of liver and kidney function may slightly differ each trimester. Blood specimens were taken on availability of the mothers and not at a specific time during the pregnancy. Not taken into account are factors such as diet and co-morbidities such as diabetes and hypertension, which may also have an effect on birth outcomes. We did not perform an evaluation of metal–metal interactions to determine possible synergistic interactions. The significant associations between adverse birth outcomes and blood levels of Mn, Fe, Se, and Cd may indicate an interaction effect.

## 7. Conclusions

This study shows significant associations between liver and kidney function biomarkers, gestational age, and Apgar score. We did not find any significant association between heavy metals Pb and Hg on ABOs at these concentrations. We did, however, find a significant association between Cd and gestational age. More research is needed to determine specific relationships between ABO and liver and kidney function biomarkers. Better-regulated bio-specimen collection during pregnancy will enable us to better compare variations in blood levels during gestation. Adding a control group and measuring more sensitive markers may contribute to a better understanding of this topic. 

## Figures and Tables

**Table 1:** Distribution of sociodemographic characteristics of participants.

	(*n* = 400)	
Paramater	Category	*n* (%)
Region	Paramaribo, Wanica, Commewijne, Saramacca, Para	298 (74.5)
	Nickerie, Coronie	55 (13.8)
	Marowijne, Brokopondo, Sipaliwini	47 (11.8)
Maternal age at intake	16–19 years	40 (10.0)
	20–34 years	301 (75.3)
	35+ years	59 (14.8)
Ethnicity *	African descent	187 (46.8)
	Asian descent	102 (25.5)
	Other	110 (27.5)
Education	No or primary	72 (18.0)
	Lower vocational or secondary	148 (37.0)
	Upper secondary/vocational or tertiary	179 (44.8)

* Ethnicity: African descent (Creole, Tribal), Asian descent (Hindustani, Javanese), Other (Caucasian, Indigenous, Mixed).

**Table 2:** Median values and interquartile ranges for liver and kidney functions and metals and elements.

Biomarker/Element	Median	IQR	Reference or Normal Concentration
Creat (umol/L)	49.00	13	44–97
Ur (mmol/L)	2.50	0.7	1.8–7.1
CysC (µg/mL)	0.62	0.17	0.57–1.79
AST (U/L)	12.00	5	15–41
ALT (U/L)	9.00	3	7–52
GGT (U/L)	11.00	8	7–50
Bl Al (µg/L)	11.90	10.74	1–3
Bl Mn (µg/L)	15.14	9.78	9.5
Bl Fe (µg/dL)	395,520.34	92,941.74	50–212
Bl Se (µg/L)	193.02	46.89	125–163
Bl Cd (µg/L)	0.26	0.18	0.315
Bl Sn (µg/L)	0.67	0.63	^#^
Bl Hg (µg/L)	2.86	2.72	0.6–0.9
Bl Pb (µg/dL)	1.98	1.94	≥5 *

* Action level. ^#^ We found no acceptable reference range or normal range in the literature.

**Table 3:** Weight regression results for birthweight, gestational age, and Apgar score.

		Gestational Age		Birthweight		Apgar Score at 5 min	
	Parameter	Beta	*p*-Value	Beta	*p*-Value	Beta	*p*-Value
Kidney	Blood Cr	0.009	0.522	0.002	0.441	<0.0001	0.937
	Blood Ur	0.131	0.569	−0.04	0.220	0.427	0.002
	Blood CysC	1.412	0.006	0.072	0.381	−0.640	<0.0001 *
Liver	Blood AST	−0.111	0.0004 *	0.004	0.299	−0.060	0.004 *
	Blood ALT	0.181	<0.0001 *	>−0.001	0.909	0.0412	0.044
	Blood GGT	0.028	0.142	−0.001	0.668	>−0.0001	0.975
	Blood Al	<0.001	0.99	<0.0001	0.039 *	−0.0169	0.004 *
Metals	Blood Mn	0.051	<0.0001 *	−0.001	0.677	−0.007	0.398
	Blood Fe	<0.001	0.0001 *	−<0.001	0.923	>−0.001	<0.0001 *
	Blood Se	−0.025	<0.0001 *	>−0.001	0.554	0.003	0.057
	Blood Cd	5.454	<0.0001 *	−0.050	0.721	0.464	0.263
	Blood Sn	0.010	0.632	0.006	0.222	−0.006	0.631
	Blood Hg	−0.027	0.515	0.007	0.222	−0.025	0.076
	Blood Pb	0.049	0.197	−0.011	0.166	0.020	0.354
Region ^#^	Rural	0.031	0.906	−0.201	0.0006 *	−1.033	<0.0001 *
	Interior	2.131	<0.0001 *	0.078	0.229	−0.030	0.853
	Urban	0		0		0	
Maternal Age	16-19	−1.338	<0.0001 *	−0.108	0.052	0.666	0.001 *
	35+	−1.283	0.0005 *	0.004	0.946	−0.231	0.143
	20–34	0		0		0	
Ethnicity	Asian	−0.255	0.513	0.132	0.024	0.021	0.920
	Else	0.664	0.006	0.072	0.099	0.254	0.087
	African	0		0		0	
Education	Secondary	−0.194	0.616	−0.031	0.606	−0.477	0.0003 *
	Tertriary	0.243	0.589	−0.029	0.689	0.074	0.722
	No/primary	0		0		0	

* Significant findings. ^#^ Region: urban: (Paramaribo, Wanica, Commewijne, Saramacca, Para); rural: (Nickerie, Coronie); interior: (Marowijne, Brokopondo, Sipaliwini).

## Data Availability

The data presented in this study are available upon request after approval of the CREEOH research team and permissions of the ethical boards.

## Supplementary Materials

The following supporting information can be downloaded at: https://www.mdpi.com/article/10.3390/jox14030059/s1, Table S1. Logistic Regression for SGA.
