# Supplementary material for: Association between Liver and Kidney Function and Birth Outcomes in Pregnant Surinamese Women Exposed to Mercury and Lead in the Caribbean Consortium for Research in Environmental and Occupational Health (CCREOH) Environmental Epidemiologic Cohort Study
Source: J Xenobiot. 2024 Aug 1;14(3):1051–63. doi: 10.3390/jox14030059 (PMC11348017; doi:10.3390/jox14030059)
Supplement: Supplementary file 1 [file jox-14-00059-s001.zip › jox-2979871-supplementary.pdf]

Journal of *Xenobiotics*

MDPI

Table S1. Logistic Regression for SGA.

	Parameter		β	Odds Ratio Estimates	Confidence Limits	
	Intercept		-5.3406000			
	BloodCr		-0.0181000	0.9820000	0.9430000	1.0220000
	BloodUr		0.4899000	1.6320000	0.9530000	2.7940000
	BloodAST		0.0974000	1.1020000	0.9990000	1.2160000
	BloodALT		-0.1229000	0.8840000	0.7890000	0.9910000
	BloodGGT		0.0324000	1.0330000	0.9860000	1.0820000
	BloodCysC		1.1246000	3.0790000	0.5390000	17.5930000
	BloodAl		-0.0272000	0.9730000	0.9410000	1.0070000
	BloodMn		0.0323000	1.0330000	0.9820000	1.0860000
	BloodFe		0.0000059	1.0000000	1.0000000	1.0000000
	BloodSe		-0.0021500	0.9980000	0.9870000	1.0090000
	BloodCd		1.4596000	4.3040000	0.3370000	55.0370000
	BloodSn		0.0165000	1.0170000	0.9240000	1.1190000
	BloodHg		-0.0644000	0.9380000	0.8240000	1.0670000
	BloodPb		-0.0600000	0.9420000	0.8040000	1.1030000
Region#	Rural	2	0.5040000	2.8080000	0.9980000	7.9000000
	Interior	3	0.0244000	1.7380000	0.4060000	7.4330000
	Urban	1				
Maternal Age	16-19	1	0.8867000	2.1100000	0.7590000	5.8700000
	35+	3	-1.0265000	0.3120000	0.0960000	1.0130000
	20-34	2				
Ethnicity	Asian	2	-0.0576000	0.5560000	0.1990000	1.5560000
	Else	3	-0.4712000	0.3680000	0.1470000	0.9220000
	African	1				
Education	Secondary	2	0.1059000	1.2640000	0.4360000	3.6660000
	Tertriary	3	0.0223000	1.1620000	0.3060000	4.4180000
	No/primary	1				

#Region Urban: (Paramaribo, Wanica, Commewijne, Saramacca, Para); Rural: (Nickerie, Coronie); Interior: (Marowijne, Brokopondo, Sipaliwini).
